# Adrenal Insufficiency: A Forgotten Diagnosis in HIV/AIDS Patients in Developing Countries

**DOI:** 10.1155/2019/2342857

**Published:** 2019-06-23

**Authors:** David D. Nassoro, Mkhoi L. Mkhoi, Issa Sabi, Alfred J. Meremo, Paul S. Lawala, Issakwisa Habakkuk Mwakyula

**Affiliations:** ^1^Department of Internal Medicine, Mbeya Zonal Referral Hospital, Mbeya, Tanzania; ^2^Department of Internal Medicine, University of Dar es Salaam, Mbeya College of Health and Allied Sciences, Mbeya, Tanzania; ^3^Department of Microbiology and Immunology, School of Medicine & Dentistry, College of Health Sciences, The University of Dodoma, Dodoma, Tanzania; ^4^National Institute for Medical Research, Mbeya Medical Research Center, Mbeya, Tanzania; ^5^Department of Internal Medicine, School of Medicine & Dentistry, College of Health Sciences, The University of Dodoma, Dodoma, Tanzania; ^6^Department of Psychiatry and Mental Health, University of Dar es Salaam, Mbeya College of Health and Allied Sciences, Mbeya, Tanzania; ^7^Department of Psychiatry and Mental Health, Mbeya Zonal Referral Hospital, Mbeya, Tanzania

## Abstract

Adrenal insufficiency (AI) is one of the most common endocrine disease in patients with HIV/AIDS, leading to high morbidity and mortality in HIV patients who become critically ill. Various etiologies are associated with the condition, including cytomegalovirus (CMV),* Mycobacterium tuberculosis*, lymphoma, Kaposi's sarcoma, and drugs such as rifampin, among others. HIV patients with advanced disease develop relative cortisol deficiency largely due to the reduction of cortisol reserve, which predisposes patients to adrenal crisis in periods of stress or critical illness. The prevalence of AI in HIV/AIDS patients during HAART era is higher in developing than developed countries, probably due to limited access to both diagnosis and adequate treatments which increases the risk of opportunistic infections. The clinical features of functional adrenal insufficiency in HIV/AIDS patients can be masked by various infectious, noninfectious, and iatrogenic causes, which reduce clinical recognition of the condition. Development of simple screening algorithms may help clinicians reach the diagnosis when approaching these patients. In many low-income countries, most HIV patients are diagnosed with advanced disease; thus, further research is necessary to elucidate the prevalence of adrenal insufficiency in HIV/AIDS patients and the condition's impact on mortality in this population.

## 1. Background

Adrenal insufficiency (AI) is one of the most common endocrine diseases in patients with Human Immunodeficiency Virus/Acquired Immunodeficiency Syndrome (HIV/AIDS), leading to high prevalence of functional adrenal insufficiency in critically ill patients. This eventually leads to a high mortality rate in patients who develop adrenal crisis and receive inadequate intervention [1, 2]. This high prevalence is attributed both to the overall condition of HIV/AIDS and its specific pathological effects upon the adrenal glands. According to WHO, HIV infection is an infection caused by Human Immunodeficiency Virus diagnosed by the criteria outlined by WHO [3], with the first screening test developed in 1984 [4] while HIV advanced disease (for individuals ≥five years) is defined as a CD4 cell count <200 cells/mm3 or a WHO clinical stage 3 or 4 event at presentation for care; however, all children with HIV younger than five years old should be considered as having advanced disease at presentation [5].

The commonest opportunistic etiology in HIV patients with AI is cytomegalovirus (CMV), whose histological prevalence can be as high as 93% in those who are not on antiretroviral therapy (ART) [6]. Other infectious etiologies include infections with* Mycobacterium avium* Complex,* Mycobacterium tuberculosis, Toxoplasma gondii, Pneumocystis jiroveci*,* Histoplasma capsulatum*, and* Cryptococcus neoformans. *Noninfectious etiologies include tumors such as lymphoma and Kaposi's sarcoma, adrenal hemorrhage (especially Waterhouse–Friderichsen syndrome), drugs such as rifampin, ketoconazole, and phenytoin, and autoimmune adrenalitis [7, 8].

Patients with HIV/AIDS tend to have functional adrenal insufficiency as they generally retain adequate or even higher secretion of cortisol, albeit at relatively higher levels of adrenocorticotropic hormone (ACTH) [9]. This can be explained with certainty that anterior pituitary function is usually maintained in an HIV patient as a evidenced by studies that used hypothalamic releasing hormone to assess its response [10, 11]. However poststimulation peak levels of ACTH may be increased in some patients, suggesting an enhanced pituitary responsiveness due to target organ dysfunction (i.e., decreased levels of serum cortisol from the adrenal glands [12]). In spite of the majority of patients presenting with some chronic symptoms of AI, other acute symptoms tend to manifest in periods of stress where there is usually an increase demand for cortisol and the production is insufficient, which can cause adrenal crisis.

Some studies showed that, during early HIV infection, adrenal stimulation with synthetic ACTH (cosyntropin test) showed inadequate response in up to 14% of the patients [9, 11], while the propotion was up to 54% in patients with advanced HIV/AIDS. This implies that there is progressive glandular dysfunction with more advanced disease [13].

## 2. Pathophysiology

### 2.1. HIV and Adrenal Insufficiency

Due to its complex interaction with the immune system, the hypothalamic-pituitary-adrenal (HPA) axis dysfunction is proportional to the level of immunodeficiency in HIV-infected patients, with greater severity noted in patients that are not on antiretroviral therapy (ART) or failing their regimens [7, 14]. In a significant number of HIV patients, increased basal cortisol level has been observed, and this underpins the state of chronic inflammation that infection creates [13]. With the advancement of HIV disease, there is also increased ACTH secretion, which indicates activation of corticotropin releasing hormone (CRH) [15]. With chronic inflammation, inflammatory mediators such as IL-1 and IL-6 together with TNF-*α* have been shown to directly stimulate the adrenal glucocorticoid pathway [9, 16]. Furthermore, the HIV envelope protein gp120 has been found to induce HPA axis hyperactivity, mostly at the hypothalamus level [17].

Glucocorticoid resistance is also one of the manifestations found in a subset of AIDS patients and is characterized by a hyperfunctioning HPA axis, which results in hypercortisolemia and increased ACTH secretion [18]. This may be explained in part by sustained chronic inflammation induced by HIV in the adaptive immune system of the gut, as this tends to play a major part in progression to advanced disease [19]. In one study of patients with AI, the glucocorticoid affinity to its ligand was found to be reduced, and an increased glucocorticoid receptor (GR) density was also noted, which is suggestive of resistance [18]. Similar to type 2 resistant asthma, these patients exhibit elevated IL-2 and IL-4 which have been implicated in reducing glucocorticoid affinity to its ligand. In addition, the balance of glucocorticoid receptor agonists and antagonists may play a role in the development of resistance. The main agonist to the glucocorticoid receptor is the GR*α*, while GR*β* is known as the main antagonist to the actions of glucocorticoids [20]. In HIV patients, there has been noted increased expression of GR*β* as compared to GR*α* [21]. Thus these patients exhibit a subsequent resistance to glucocorticoid action [22, 23].

Hyperreninemic and hyporeninemic hyperaldosteronism have all been found in HIV-infected patients and may lead to electrolyte imbalance [24]. While frank hypoaldosteronism is uncommon, an impaired aldosterone response to ACTH has been observed in many HIV-infected patients [25]. Electrolyte imbalances are commonly found in AIDS patients, but these abnormalities are usually due to adverse drug events and syndrome of inappropriate antidiuretic hormone secretion (SIADH) mainly due to respiratory and CNS infections like Pneumocystis Jiroveci Pneumonia, commonly called PCP and Central nervous system toxoplasmosis, and hence may not necessarily be due to hyperaldosteronism [26]. Two cases of primary hyperaldosteronism have been reported in HIV-infected patients, whereby the proposed mechanism was a renin-like activity of HIV aspartic protease [27].

In HIV/AIDS patients there is also a significant shift to cortisol synthesis at the expense of androgens [28]. Such a pattern has been observed in both acute and chronic illness, as well as among malnourished patients [29]. This may be due to the decreased overall activity of the 17, 20- lyase enzyme, resulting in reduced Dehydroepiandrosterone (DHEA) production. Given DHEA is the precursor to androgens, this process results in diminished androgen levels [30]. Decreased DHEA production also marks in the preference toward glucocorticoid synthesis at the expense of androgen synthesis. In women with AIDS-related wasting syndrome, the DHEA to cortisol ratio is significantly decreased while ovarian androgen response after Human chorionic gonadotropin (hCG) stimulation is usually normal [30, 31].

### 2.2. Infectious Etiologies

Infectious etiologies are generally the most common causes of AI in developing countries where the infectious manifestation of the adrenals includes a diverse array of viruses, fungi, and bacteria [32]. Immunocompromised individuals such as those with HIV/AIDS are at greatest risk for either primary adrenal infection or disseminated microbial disease involving the adrenal glands [33]. Among the commonest infectious etiologies in HIV/AIDS patients are Cytomegalovirus and Tuberculosis [6].

With advanced immunosuppression, the risk of disseminated Cytomegalovirus and Mycobacterium pathogens is quite high with multisystemic involvement including the suprarenal glands. Studies in AIDS patients and transplant recipients indicate that the CD-4 dependent cytotoxic T lymphocyte activity of CMV antigen-specific CD8+ T cells is critical for preventing CMV replication and end-organ disease. Furthermore, it has been shown that CD4 cell function and/or number is important in preventing uncontrolled CMV replication [33, 34]. The risks of developing Disseminated CMV infection in HIV patients other than AIDS is high in males (71%), Malignancy (57%) and chronic renal failure (43%) [35]. Despite being one of the commonest causes of iatrogenic AI, supraphysiological doses of glucocorticoids, especially in AIDS patient during treatment of conditions such as Pneumocystis Pneumonia, have also been associated with adrenal crisis secondary to CMV adrenalitis probably by causing viremia [36, 37]. In an autopsy study that assessed the pathological findings in 74 AIDS cases, 31 had CMV involvement of the adrenal glands while CMV adrenalitis was diffuse in 10 cases and focal manifestation was observed in 20 cases. The inflammatory infiltrate was predominantly polymorphonuclear leukocyte admixed with some lymphocytes. Varying degree of fibrosis was encountered in 60% of cases with diffuse fibrosis in 17% of cases [38]. Usually when 90% or more of the pair of glands has been destroyed, clinical features of AI unravels [39]

Clinical features of AI secondary to tuberculous adrenalitis are generally more apparent in HIV patients with advanced disease specifically with a CD4 cell count of fewer than 100 cells/mm^3^ [40, 41]. Similar to other tissues, Tuberculosis of the adrenals leads to inflammation, necrosis with eventual progressive destruction of cortical tissue [42]. Furthermore, studies have shown that disseminated tuberculosis, whether hematogenous, or lymphatic from the primary area of infection is the source of adrenal infiltration [43]. The definite tropism of Tubercle bacilli to the adrenals is still mysterious.

### 2.3. Other Pituitary Axes

The gonads and growth hormone pituitary axes are some of the most commonly affected in HIV patients. Despite normal response to gonadotropin releasing hormone, hypogonadotropic hypogonadism tends to occur quite frequently especially in AIDS patients primarily due to hypothalamic dysfunction [44]. Sexual hormone binding globulin levels are frequently increased in HIV-positive men and levels of testosterone are usually normal; however, levels of free testosterone may be low [45]. The abnormality may be due to various factors, the main one being a chronic systemic illness that is mainly caused by HIV itself with one of the outcomes being suppression of the hypothalamic-pituitary-gonadal axis [19]. The prevalence appears to be higher in patients with advanced disease ranging from 30% to 80% in some cases [46, 47] and since the introduction of HAART in the early stages of the disease, the prevalence has fallen to around 20% as seen in some studies [48, 49].

HIV infection does not appear to affect Growth hormone (GH) circadian pulse as evident from samples of asymptomatic HIV-infected and clinically stable AIDS patients [50]. Despite normal GH pulse frequency and insulin-like growth factor (IGF)-1 levels, lower mean GH concentrations, basal GH concentrations, and GH pulse amplitude are observed on patients with HIV associated lipodystrophy [50]. Also reduced GH levels appear to be inversely related to visceral obesity in HIV associated lipodystrophy [50] with up to a third showing biochemical deficiency [51]. Currently, the clinical implication of this growth hormone deficiency remains unclear and is less severe than that in patients affected by growth hormone deficiency secondary to pituitary disease.

## 3. Adrenal Insufficiency in Pre- and during HAART Era

From the late 1930s until the emergence of HIV in the early 1980s, the commonest documented etiology of AI was tuberculosis and the rapid progression were mostly linked to tuberculous adrenalitis as well [52, 53]. Few etiologies other than tuberculosis were known during this time; this may be because there were no clear temporal relationships to link the condition to other causes probably due to the unavailability of diagnostic test with high specificity and sensitivity during this period.

Since its description in 1981 until 1987 when the first ART, zidovudine introduced, being diagnosed with HIV was synonymous to a death sentence, there was no treatment with significant impact to the progression of the disease [54]. Furthermore, during that era, a significant proportion of HIV-infected patients developed AIDS as compared to HAART era [55, 56]. With the commencement of the first monotherapy with zidovudine, resistance started to develop quickly especially in HIV patients with advanced disease and hence a significant proportion of patients continue to deteriorate as a result of treatment failure [57]. Therefore, with advanced disease being the major risk factor for the development of AI, the prevalence of the condition was significantly higher during the pre-HAART era as compared to the HAART era.

With functional AI, clinical manifestation tends to manifest later when the patient has advanced disease with or without critical illness. Perhaps this leads to underdiagnosis of the condition during the pre-HAART era one of the reasons being that clinical features of the advanced disease are quite similar to chronic form of AI. One of the earliest studies to alert the medical community about the condition was by Tapper et al. in 1984 where, during the autopsy of ten AIDS patient, all of them had significant glandular inflammatory changes of the adrenal glands secondary to cytomegalovirus [58]. In the same year, another study that used synthetic ACTH published by Greene et al. showed the prevalence to be 20% [52]. Similar findings were observed by Membreno et al. in 1987 [59]. The low prevalence may be explained by the fact that supraphysiological doses of synthetic ACTH were used and are usually associated with false negatives especially in patients with functional AI. There is a paucity of data in developing countries during the pre-HAART era.

HAART avoids opportunistic infections and consequently, the prevalence of AI dropped dramatically and is now quite rare in developed countries. At the same time, limited diagnosis and adequate treatments lead to development of AI in a consistent number of cases in low-income countries where majority of patient delay seeking treatment until the disease is relatively advanced [60]. Furthermore, mostly in developing countries, engagement in risky behaviors such as unprotected sexual intercourse and multiple sexual partners continues to maintain the steady state of HIV transmission.

According to UNAIDS 2018 Global HIV and AIDS statistics, in many developed countries, specifically in North America and western and central Europe, the number of new HIV infection has been falling quite significantly falling by 8% from the year 2000 to 2017. Also, during the same time, the number of AIDS-related deaths fell by 36% and incidence to prevalence ratio has fallen to 0.03 from more than 0.09 in the period from 1990 to 2017. Furthermore, AIDS-related mortality has decreased from more than 80,000 to around 13,000 in the period from 1990 to 2017 [61]. The data highlights the extent to which developed countries have been able to control the HIV epidemic in the past three decades.

In developed as compared to developing countries, a significant number of HIV patients know their status, are on treatment, and are virally suppressed as compared to developing countries [62–64]. This may explain the low prevalence of patients with advanced disease in developing countries during the HAART era and hence the expected low prevalence of AI. During the HAART period, studies that assessed AI in HIV/AIDS patient in developed countries were expectedly few.

The biochemical presentation of adrenal insufficiency is quite common in hospitalized AIDS patients (17%) as compared to nonhospitalized symptomatic patients (4%) [1, 65]. Studies that assess adrenal insufficiency's prevalence and level of HPA axis dysfunction in critically ill HIV/AIDS patients in developing countries are few.

In 2017, a study done in Pakistan to evaluate AI using high dose cosyntropin among treatment-experienced patients attending treatment centers found the prevalence to be 14% [66]. However, the prevalence may not be truly representative of treatment-naïve or experienced patients, as there was no analysis of how many patients were on treatment and how many were not. Furthermore, the study did not report the proportion of patients who had early or advanced disease.

A study done in India in 2012 found that the prevalence of AI among AIDS patients, as identified with the use of low dose ACTH stimulation testing, was 76% [67]. In the study, the prevalence was higher probably because of selecting patients with advanced disease as opposed to all patients that were seropositive for HIV. Moreover, because of high prevalence of AI among AIDS patients, low dose synthetic ACTH expectedly had higher sensitivity and specificity as compared to higher doses.

A study in Nigeria by Odeniyi et al. found the prevalence of 34.8% in treatment naïve patients [68]. In this study, the proportion of patients with AIDS was not reported, and hence the high prevalence observed cannot be clearly explained. Furthermore, in HIV/AIDS patients, the proportion of patients with tuberculous adrenalitis that develop clinical features of AI is not known. This may be affected by the high costs of diagnosis [69] as for infectious etiologies, biopsy of the adenals is may be important.

In 2007, a study done in Uganda by Meya et al. found the prevalence of functional adrenal insufficiency in critically ill patients with HIV/AIDS to be 19% [70]. This prevalence is consistent to other studies as previously discussed. Data regarding the prevalence of AI in HIV-infected and uninfected patients in other East African countries, including Tanzania, is significantly lacking partly because the diagnostic test (synthetic ACTH) is usually unavailable or prohibitively expensive, with costs approximately USD 100 for a single test. Furthermore, limited availability of laboratory services particularly in remote areas may partly account to rarity of this disease condition among HIV/AIDS patients' comorbidities in Tanzania.

## 4. Clinical Presentation

There are many clinical features that may lead to a suspicion of AI in an HIV patient, but most of the time it may prove difficult to attribute them directly to the suprarenal gland's dysfunction. The most common clinical features in these patients are fatigue (84-95%), weight loss (66-76%), other gastrointestinal symptoms such as nausea, vomiting, and abdominal pain (49-62%), and muscle and joint pain (35-40%) [71–73]. These presentations are quite similar to the ones found in patients with tuberculosis, which is quite prevalent in HIV patients, especially considering that the chronic form of AI in advance stages may also present with low grade fever [74].

In regions with high prevalence of tuberculosis, like sub-Saharan Africa, it may be difficult for the clinician to suspect AI as a diagnosis or even consider it on a differential, even though the chances of having disseminated tuberculosis, which includes mycobacterial infiltration of the adrenal glands, are significantly higher in HIV patients with advanced disease. Therefore, it is imperative to have a high degree of suspicion in these patients, especially if they have concomitant tuberculosis or other opportunistic conditions associated with advanced disease, such as disseminated cryptococcosis, cytomegalovirus, and pneumocystis.

Other signs that are more specific to primary adrenal insufficiency include skin hyperpigmentation (41-74% of patients), postural hypotension (55-68% of patients), and salt craving (38-64% of patients) [71].

Antiretroviral drugs like zidovudine have been shown to produce hyperpigmentation and hence mimic features of primary AI [75]. Other drugs that may cause hyperpigmentation include tetracyclines, some antimalarials such as chloroquine and hydroxychloroquine, and phenothiazines such as chlorpromazine, promethazine, and fluphenazine.

Commonest laboratory findings in AI patients include hyponatremia (70-80%), hyperkalemia (30-40%), and anemia (11-15%) [71]. However, it is important to note that low or normal potassium levels may be observed in HIV patients with either diarrhea or vomiting [76]. Hyponatremia may be explained by SIADH, which may be secondary to PCP or central nervous system toxoplasmosis, additionally reducing the suspicion of AI [77, 78]. In addition to its SIADH-inducing effect, high doses of cotrimoxazole used in treatment of PCP have also been found to produce a dose-dependent natriuretic effect, resulting in hyponatremia. [79]. Even though uncommon, secondary and tertiary AI may manifest without hyperpigmentation, hyperkalemia, or gastrointestinal symptoms. Also, patients may present with hypoglycemia in the absence of dehydration [23, 80]. In contrast, hypotension is a clinical feature suggestive of AI with a high degree of specificity but is usually present in the setting of adrenal crisis, a condition in which mortality is usually high especially in the HIV-positive patient.

## 5. Diagnostic Tests

There are various tests that have been developed for screening and diagnosis of adrenal insufficiency. Serum ACTH and cosyntropin stimulation test are the most commonly used diagnostic tests for AI. Cosyntropin is an ACTH analogue with the same physiologic action as corticotropin. Other tests, such as insulin-induced hypoglycemia and the metyrapone test, can be somewhat cumbersome and relatively difficult to perform. In the former, even though the test has high sensitivity comparable to high dose cosyntropin, there is always a risk of hypoglycemia which may prove detrimental especially in critically ill HIV patients. The latter involves the use of metyrapone, which blocks cortisol biosynthesis by blocking the 11*β*-hydroxylase enzyme, the last step in the synthesis of cortisol, which leads to accumulation of 11-deoxycortisol.

Serum cortisol is one of the tests that can be used more in screening than in the diagnosis of AI. In patients with an intact HPA axis, serum cortisol ranges between 10 and 20 mcg/dl early in the morning (~6 AM). An early morning cortisol concentration of less than 3mcg/dl is highly suggestive of AI [81–83]. However, it has been found that early morning values of cortisol less than 5mcg/dl had almost 100 percent specificity and 36 percent sensitivity. Likewise, when using a higher serum titer of 10mcg/dl as a criterion for AI, this increased the sensitivity to 62 percent but reduced the specificity to 77 percent [84, 85]. Given the low sensitivity of serum cortisol testing, even with raising the cutoff level, serum cortisol testing does not contribute significantly to the definitive diagnosis of AI.

Cosyntropin stimulation test is the test of choice for the diagnosis of AI. For healthy individuals, cortisol response is greatest early in the morning. In contrast, in patients with AI, especially those with significantly reduced adrenal hormone reserve, the response is the same even if tested in the afternoon [86, 87]. This is due to a reduction in cortisol reserves in zona fasciculata that results in persistently lower peak response. A high dose (250 mcg) ACTH stimulation test is typically preferred in patients with primary AI and most patients with secondary AI [88], while low dose (1 mcg) is preferred in patients with suspected secondary, tertiary, or in some cases, functional AI [89, 90].

## 6. Clinical Implication

In many developing countries like Tanzania, AI is generally diagnosed using clinical features backed up with some nonspecific laboratory investigations which may have low sensitivity and/or specificity. Serum cortisol, sodium, potassium, and ACTH are among the commonly available tests; however they are of little help in diagnosis and furthermore they frequently run out of stock or not available at all. Despite highly prohibitive testing cost for AI most HIV/AIDS patients tend to present late and hence at risk of opportunistic infections that may accompany adrenal crisis and hence will depend on the clinician's high index of suspicion to reach the diagnosis to prevent deaths associated with AI. Therefore, we recommend that screening should be in HIV patient that are not or failing on HAART and presents with suggestive clinical features and on the other hand, it is less common and should not be routinely checked in treated patients with suppressed viral load and with early disease in the absence of clear signs or symptoms.

## 7. Opportunities and Challenges

Globally and especially in the developing world, the uptake of HIV testing in the general population is poor. In US about two in five newly diagnosed HIV patients presented with WHO stage 3 or 4 disease condition [91]. Similarly, in Uganda and South Africa, up to 40% of the newly diagnosed HIV patients present at stages 3 and 4 [92, 93] while in Tanzania more than 60% of patient with HIV have late presentation and 40% of these patients have CD4 counts less than 200 cells/mm^3^ [94]. This late presentation of newly diagnosed HIV/AIDS increases the chances of adrenal gland infiltration and reduction of its hormonal reserve.

Tanzania has one of the highest prevalence rates of and mortality rates from tuberculosis/HIV coinfections, according to one meta-analysis [95]. Tuberculosis is also the most common reason for hospital admission among HIV patients in Tanzania. With disseminated tuberculosis being the second most common cause of AI among this patient population [96], it is imperative that patients suspected to have severe form of tuberculosis are screened for AI. Although this approach may translate to heightened prevalence of AI in patients with HIV/AIDS as compared to the general population, the need to avoid adverse complications associated with AI among this patient population is undoubtedly crucial. Thus, further research studies are needed to help better define the prevalence of AI among HIV/AIDS patients and its contribution to mortality.

## 8. Conclusion and Recommendations

AI is among the common endocrinological manifestation in patients with HIV/AIDS. In developing countries, almost half of all newly diagnosed HIV cases have advanced disease and furthermore, more than 50% of HIV hospital admissions are in-patients with advanced disease, with majority of these patients having TB/HIV coinfection. With prevalence of AI in patients with advanced HIV disease reaching up to 50%, it is concerning that AI may be a neglected but significant contributor of morbidity and mortality in this population. Contributing to this challenge may be difficulties with laboratory diagnosis or even clinical suspicion of the condition, especially given the fact that its clinical presentation may overlap with that of other disease conditions or adverse drug events in an HIV/AIDS patient. Hence, clinicians need to maintain a high degree of suspicion when managing patients with risk for AI. This not only will improve timely diagnosis and treatment of these patients, but also may decrease their morbidity and mortality as well. Further research on AI in HIV patients will be necessary to elucidate its prevalence and assess its impact on mortality in this patient population.
